# Special Issue: “New Trends in Diabetes, Hypertension, and Cardiovascular Diseases—2nd Edition”

**DOI:** 10.3390/ijms26020449

**Published:** 2025-01-07

**Authors:** Yutang Wang, Dianna J. Magliano

**Affiliations:** 1 Discipline of Life Science, Institute of Innovation, Science and Sustainability, Federation University Australia, Ballarat, VIC 3353, Australia; 2 Diabetes and Population Health, Baker Heart and Diabetes Institute, Melbourne, VIC 3004, Australia

## 1. Introduction

Cardiovascular diseases (CVDs) encompass a range of conditions affecting both the heart (e.g., coronary heart disease and heart failure [1]) and blood vessels (e.g., cerebrovascular disease [2] and peripheral artery disease [3]). CVDs remain the leading global cause of death, claiming approximately 17.9 million lives annually [4]. Beyond their toll on human lives, CVDs impose a significant economic burden, with annual costs exceeding USD 400 billion. This is projected to rise to USD 1.49 trillion by 2050 in the United States alone [5,6], underscoring the urgent need for further research to develop cost-effective treatments, preventive programs, and policies to enhance cardiovascular health [7].

Hypertension and diabetes are common co-occurring conditions in individuals with CVDs [8,9,10], affecting 31.1% and 8.5% of the adult population, respectively [11,12]. Both conditions serve as independent risk factors for CVDs [13,14], and, thus, their effective management is central in CVD treatment strategies [15,16,17].

Oxidative stress and inflammation are well-established mechanisms underlying both hypertension [18] and diabetes [19,20]. The onset of these conditions is closely linked to a disruption in the balance between oxidants and antioxidants marked by the excessive production of reactive oxygen species (ROS) and a compromised antioxidant defense system. Elevated ROS levels foster inflammation, damage key molecules such as DNA, proteins, and lipids, and lead to cellular and tissue impairment, which contributes to the development of hypertension and diabetes [18,19].

Similarly, oxidative stress and inflammation play critical roles in the development of atherosclerosis [21], the primary pathological driver of CVDs [22,23]. ROS oxidize low-density lipoprotein (LDL), and the oxidized LDL subsequently triggers inflammation and promotes atherogenesis [24].

Animal models are indispensable in investigating the pathogenesis of CVDs and developing therapeutic interventions [25]. A variety of species, including mice, rats, rabbits, goats, pigs, and primates, have been employed as models for CVD research [26,27,28,29]. Among these, rodent models are especially valuable due to their close resemblance to humans in terms of cardiovascular conditions, their high reproductive capacity, and the ease of genetic modification [30]. Each animal model has unique advantages and limitations, so selecting the most appropriate model that closely mimics the relevant human cardiovascular condition is essential in advancing research [31].

CVD management typically involves a combination of medications, surgeries, and lifestyle modifications [32,33,34]. A wide range of drugs are used to treat CVDs, including anticoagulants, antiplatelet agents, angiotensin-converting enzyme inhibitors, angiotensin II receptor blockers, beta blockers, calcium channel blockers, cholesterol-lowering agents, diuretics, and vasodilators [35]. A novel approach to CVD treatment, known as targeted therapy, aims to treat disease-causing cells or molecules (such as specific proteins or genes) without affecting normal tissue, enabling personalized and precise medicine [36,37]. Current targeted therapies include protein drugs, nucleic acid-based therapies, gene-editing technologies, and cell-based treatments [36].

In addition to pharmacological interventions, adopting a healthy diet represents a key lifestyle change that can reduce CVD risk [33]. This can include (1) an increased fruit, vegetable, legume, whole grain, nut, and fish intake [38]; (2) replacing saturated fats with monounsaturated and polyunsaturated fats [39]; (3) a reduced sodium and cholesterol intake [40]; (4) minimizing processed meats, refined carbohydrates, and sugary beverages [41]; and (5) avoiding trans fats [42].

Regular physical activity is another vital component of lifestyle modifications to lower CVD risk [43,44]. Current guidelines recommend that adults engage in at least 150 min of moderate-intensity exercise or 75 min of vigorous-intensity exercise per week to reduce the risk of CVDs [33].

## 2. Contributions of This Special Issue to This Field of Research

This Special Issue presents the latest research on diabetes, hypertension, and CVDs, offering valuable insights into current advancements in these fields, for instance, increasing our understanding of recent developments in lifestyle interventions for CVD management. A notable contribution by Napiórkowska-Baran et al. [45] provides a comprehensive review of how various nutrients—such as macronutrients, micronutrients, and vitamins—can modulate inflammation and immune function, and how they may help protect against both CVDs and diabetes. Additionally, Tan et al. [46] explored the effects of exercise in a rat model, revealing that exercise alleviated glucose intolerance, cardiac inflammation, and adipose tissue dysfunction. These findings align with previous studies [47,48], collectively confirming that exercise is an effective strategy in reducing the risk of CVDs and diabetes [49,50].

This Special Issue also highlights recent developments in other areas, including animal models, risk factors, novel diagnostic techniques, and disease mechanisms (e.g., epigenetic regulation and inflammation). The articles cover a range of disease conditions, such as glucose intolerance, diabetes, hypertension, endocarditis, and heart failure, providing valuable insights into disease pathogenesis and potential therapeutic strategies. We encourage readers to explore these articles in detail, to gain a deeper understanding of each topic [25,45,46,51,52,53,54,55,56,57,58] (Table 1).

## 3. Future Research Directions

There are several promising directions for future research, offering significant potential. These include precision medicine [59], the identification of risk factors [60] and biomarkers for early detection [61], the application of machine learning [62], the use of remote monitoring and digital health tools [34], and the exploration of the interactions between the microbiome and cardiovascular health [63]. The research in these areas is expected to become increasingly multidisciplinary, incorporating expertise from various fields. The ultimate goal of this research is to personalize cardiovascular care, reduce the global burden of CVDs, and enhance patient outcomes worldwide.

## 4. Conclusions

This Special Issue provides a comprehensive overview of recent advancements in the research on hypertension, diabetes, and CVDs, covering key aspects such as pathogenesis, diagnosis, potential treatment options, and animal models. The findings presented are likely to make a significant contribution to the ongoing efforts to reduce CVD-related morbidity and mortality.

## Figures and Tables

**Table 1:** Summary of the articles in this Special Issue.

Article Types	Title	Authors	Main Finding
Review	Immunomodulation through Nutrition Should Be a Key Trend in Type 2 Diabetes Treatment [45]	Napiórkowska-Baran, K., Treichel, P., Czarnowska, M. et al.	This review examines how various nutrients—macronutrients, micronutrients, vitamins, and other dietary components—can modulate immune function, providing insights into immune dysfunction in diabetes patients
Review	Epigenetic Regulation of the Renin–Angiotensin–Aldosterone System in Hypertension [51]	Takeda, Y., Demura, M., Yoneda, T., Takeda, Y.	This review explores the role of epigenetic mechanisms in the regulation of this system, emphasizing its significance in hypertension pathogenesis
Review	Lipoprotein (a) as a Cardiovascular Risk Factor in Controversial Clinical Scenarios: A Narrative Review [52]	Abdalla, H.M., Mahmoud, A.K., Khedr, A.E. et al.	This review investigates the impact of lipoprotein (a) on CVD development, underscoring its potential therapeutic applications
Review	Novel Diagnostic Methods for Infective Endocarditis [53]	Burban, A., Słupik, D., Reda, A. et al.	This review highlights innovative diagnostic techniques for infective endocarditis, noting their advantages in terms of speed and accuracy
Review	Animal Models, Pathogenesis, and Potential Treatment of Thoracic Aortic Aneurysm [25]	Wang, Y., Panicker, I.S., Anesi, J. et al.	This review summarizes current animal models for thoracic aortic aneurysm (TAA), discussing its pathogenesis and prospective treatment options
Review	Beyond Anticoagulation: A Comprehensive Review of Non-Vitamin K Oral Anticoagulants (NOACs) in Inflammation and Protease-Activated Receptor Signaling [54]	Jannati, S., Patnaik, R., Banerjee, Y.	This review consolidates evidence regarding the anti-inflammatory effects of NOACs, suggesting their potential role in managing inflammatory diseases
Article	T-Cell Receptor Sequences Identify Combined Coxsackievirus–Streptococci Infections as Triggers for Autoimmune Myocarditis and Coxsackievirus–Clostridia Infections in Type 1 Diabetes [55]	Root-Bernstein, R.	This study revealed that TCR sequences resembling coxsackievirus and clostridia antigens were more prevalent in type 1 diabetes, while those resembling coxsackievirus and streptococcal antigens were elevated in autoimmune myocarditis patients, providing insights into autoimmune disease mechanisms
Article	Does Hypertension Affect the Recovery of Renal Functions after the Reversal of Unilateral Ureteric Obstruction? [56]	Hammad, F.T., Lubbad, L., Al-Salam, S. et al.	This study examined the long-term effects of hypertension on renal recovery post-surgery, indicating significant impacts on renal injury and function forty-five days after the reversal of obstruction
Article	Heart Failure Promotes Cancer Progression in an Integrin β1-Dependent Manner [57]	Langier Goncalves, I., Awwad, L., Aviram, S. et al.	This research explored the relationship between heart failure and cancer progression, revealing that integrin β1 on cancer cells mediated the precancerous effects of heart failure
Article	Female *Psammomys obesus* Are Protected from Circadian Disruption-Induced Glucose Intolerance, Cardiac Fibrosis and Adipocyte Dysfunction [58]	Tan, J.T.M., Cheney, C.V., Bamhare, N.E.S. et al.	This work studied sex differences in response to circadian disruption in sand rats, finding that females exhibited protection against glucose intolerance, cardiac fibrosis, and adipocyte dysfunction
Article	Exercise Reduces Glucose Intolerance, Cardiac Inflammation and Adipose Tissue Dysfunction in *Psammomys obesus* Exposed to Short Photoperiod and High-Energy Diet [46]	Tan, J.T.M., Price, K.J., Fanshaw, S.R. et al.	This study assessed the effects of exercise on male sand rats subjected to a short photoperiod and high-energy diet, concluding that exercise alleviated glucose intolerance, cardiac inflammation, and adipose tissue dysfunction
